# Induction of RNAi Core Machinery’s Gene Expression by Exogenous dsRNA and the Effects of Pre-exposure to dsRNA on the Gene Silencing Efficiency in the Pea Aphid (*Acyrthosiphon pisum*)

**DOI:** 10.3389/fphys.2018.01906

**Published:** 2019-01-09

**Authors:** Chao Ye, Xin An, Yi-Di Jiang, Bi-Yue Ding, Feng Shang, Olivier Christiaens, Clauvis Nji Tizi Taning, Guy Smagghe, Jinzhi Niu, Jin-Jun Wang

**Affiliations:** ^1^Key Laboratory of Entomology and Pest Control Engineering, College of Plant Protection, Southwest University, Chongqing, China; ^2^Academy of Agricultural Sciences, Southwest University, Chongqing, China; ^3^Department of Plants and Crops, Faculty of Bioscience Engineering, Ghent University, Ghent, Belgium

**Keywords:** aphids, RNA interference, *hunchback*, pre-exposure, double-stranded RNA

## Abstract

The pea aphid, *Acyrthosiphon pisum*, is an important agricultural pest and biological model organism, and RNA interference (RNAi) is an important tool for functional genomics and for insect pest management. However, the efficiency of RNAi in pea aphids is variable, limiting its application in aphids. In this study, we present optimized conditions for inducing and increasing the gene silencing efficiency of RNAi in pea aphids. The optimal gene silencing of the target *Aphunchback* gene was achieved by injecting 600 ng double-stranded (ds) RNA, and the highest mRNA depletion rate (74%) was detected at 36 h after injection. Moreover, the same gene silencing conditions were used to achieve transcript silencing for nine different genes in the pea aphid, although the silencing efficiencies for the different genes varied. Furthermore, the pre-exposure of aphids to dsRNA (600 ng ds*GFP*) led to significant *hunchback* silencing following a secondary exposure to 60 ng of ds*hunchback*, a dose which did not lead to gene silencing when independently injected. The information presented here can be exploited to develop more efficient RNAi bioassays for pea aphids, both as gene functional study tools and an insect pest control strategy.

## Introduction

Over the past two decades, RNA interference (RNAi) has become a powerful tool for gene functional studies in various organisms by delivering gene-specific double-stranded RNA (dsRNA), and it is now also recognized as a next-generation insect pest control tool (Wang et al., 2017; Zhang et al., 2017). In insects, both viral infections (Marques et al., 2013; Niu et al., 2016) and non-specific exogenous dsRNAs (Flenniken and Andino, 2013; Cappelle et al., 2016) can induce the activity of the small interfering (si) RNA pathway. In brief, dsRNAs, either derived from a virus or through exogenous delivery, are processed by Dicer-2 (Dcr-2) into 21–22-bp siRNAs, while R2D2 acts as a dsRNA-binding protein. Then, these siRNAs associate with the Argonaute-2 (Ago-2) protein, to form the RNA-induced silencing complex with other proteins. Subsequently, one of the small RNA strands is selected as the guide strand and serves as a sequence-specific guide to cleave target mRNA through complementary binding. While RNAi has been successfully used in many insect species (Bellés, 2010; Tabunoki et al., 2016), RNAi efficiencies in insect pests are still variable. Some factors that may influence the efficacy of RNAi efficacy include dsRNA degradation (Singh et al., 2017), dsRNA uptake (Huvenne and Smagghe, 2010), the expression of RNAi-related genes (Garbutt and Reynolds, 2012), the transcript abundance of target genes (Scott et al., 2013) and viral infections (Swevers et al., 2013).

Aphids, agricultural pests that devastate through sap-feeding and the transmission of plant viruses, are biological models for the study of various areas, such as phenotypic plasticity, microbe–insect–plant interactions, and sexual and asexual reproduction behavior. To date, the genomes of several aphids species have been sequenced, including the pea aphid *A. pisum* (Calevro et al., 2010), *Myzus persicae* (Mathers et al., 2017), *Aphis glycines* (Wenger et al., 2017), and *Diuraphis noxia* (Nicholson et al., 2015). Their genomes revealed conserved sets of components in the siRNA pathway in comparison with other insects, but showed an expansion of microRNA pathway components (Ortiz-Rivas et al., 2012). In the model aphid, *A. pisum*, RNAi has been successfully used for functional genomics studies, including studies on the RNAi mechanism itself, and holds promise as a pest control strategy (Christiaens and Smagghe, 2014). Nevertheless, the literature suggests that the RNAi efficiency in aphids, especially through feeding, can be variable, possibly owing to the nucleolytic degradation of dsRNAs in the aphid body or suboptimal cellular uptake of dsRNAs (Christiaens et al., 2014; Cooper et al., 2018). Additionally, at present, the association between RNAi efficiency and the expression levels of core RNAi machinery components upon the delivery of the exogenous dsRNA is still unclear in aphids. Therefore, in this study, we evaluated dose- and time-dependent gene silencing efficiencies in the pea aphids, *A. pisum*, and further explored the association between RNAi efficiency and the expression levels of core RNAi machinery components, especially when aphids were pre-exposed to dsRNA.

## Materials and Methods

### Aphid Colony

In this study, the pea aphid (*A. pisum*) strain was originally collected at a *Medicago sativa* field in Gansu Agriculture University (Gansu, China) in 2012. The aphids used in this study were reared on broad bean seedlings at 22 ± 1°C, with ∼70% relative humidity and a 16-h:8-h (light:dark) photoperiod in a climate chamber. Three- to five-day-old green adults were selected for the experiments (Supplementary Figure S1).

### RNA Extraction, cDNA Synthesis and Cloning

To monitor mRNA levels of different target genes post-dsRNA injection, all experiments contained four replicates (each replicate was injected at the same time) with four individuals per replicate. Total RNA was extracted from each using the TRIzol reagent (Invitrogen, Carlsbad, CA, United States) according to the manufacturer’s instructions. The RNA was then quantified using a NanoDrop One microvolume UV-Vis spectrophotometer (Thermo Scientific, Wilmington, MA, United States). The purities of the RNA samples were assessed at absorbance ratios of OD_260/280_ and OD_260/230_, and then the integrity levels of the samples were evaluated by electrophoresis using 1% (*w*/*v*) agarose gels. The contaminating genomic DNA in the samples was removed by a DNA I treatment. The first-strand cDNA from mRNA was synthesized using a PrimerScript RT Reagent Kit (TaKaRa, Dalian, China) following the manufacturer’s protocol. The cDNA was stored at -20°C until use. The sequences of the RNAi target genes, *Aphunchback, ApAChE-1, ApCar668, ApCHS, ApC002, ApHMGR, ApJHBP, ApSid-1-like, ApSod-2*, and *ApVGSC*, as well as core RNAi machinery components, *ApDcr2, ApR2d2*, and *ApAgo2*, were retrieved from the *A. pisum* genome database. Accession numbers for these genes are provided in Supplementary Table S1. All primers for both qPCR and/or dsRNA synthesis were designed by NCBI Primer-BLAST^1^. Sequences for the primers are listed in Supplementary Table S2. The fragments of targeted genes were amplified from cDNA, prepared from whole-aphid-body RNA isolates, by PCR. The PCR conditions were as follows: initial denaturation at 95°C for 3 min, followed by 38 cycles of 30 s at 95°C, 30 s at 60°C, and 45 s at 72°C, with a final extension of 7 min at 72°C. The PCR fragments were gel-purified with a Gel Extraction Mini Kit (TaKaRa) and ligated into the pGEM-T easy vector (Promega, Madison, WI, United States). Recombinant plasmids were subsequently sequenced on an ABI Model 3100 automated sequencer (Invitrogen Life Technologies, Shanghai, China). Afterward, the plasmids were cloned in LB broth medium for 24 h in a thermostatic oscillator at 37°C and then maintained in a 4°C freezer before the subsequent synthesis of dsRNAs.

### Synthesis and Injection of dsRNA

The dsRNA was synthesized using the TranscriptAid T7 High Yield Transcription Kit (Thermo Scientific, Wilmington, MA, United States), according to the manufacturer’s instructions. The injection of dsRNA was performed using a M3301 micromanipulator (World Precision Instruments, Sarasota, FL, United States). Briefly, boiled 1% agarose was poured into a petri dish and allowed to cool until solid. Then, two cross-shaped grooves were made in the agarose for the immobilization of the aphids. Glass capillaries (3.5-in 3-000-203-G/X micropipettes, Drummond Scientific, Broomall, PA, United States) were prepared with a P-97 Micropipette Puller (Sutter Instrument, Novato, CA, United States) using the following parameters: Heat = 575, Pull = 145, VEL = 145, and DEL = 100. During the injection, the aphids were first immobilized by incubating on ice for 5 min, and then placed on the agarose plate before 120 nL of the dsRNA or water (control) was injected laterally, between the middle and hind legs of the adult aphids (Sapountzis et al., 2014). Each treatment was injected into 25 aphids. Subsequently, the aphids were moved to *Vicia faba* leaves and kept in petri dishes.

### Quantitative Real-Time PCR (qPCR)

**FIGURE 1:**
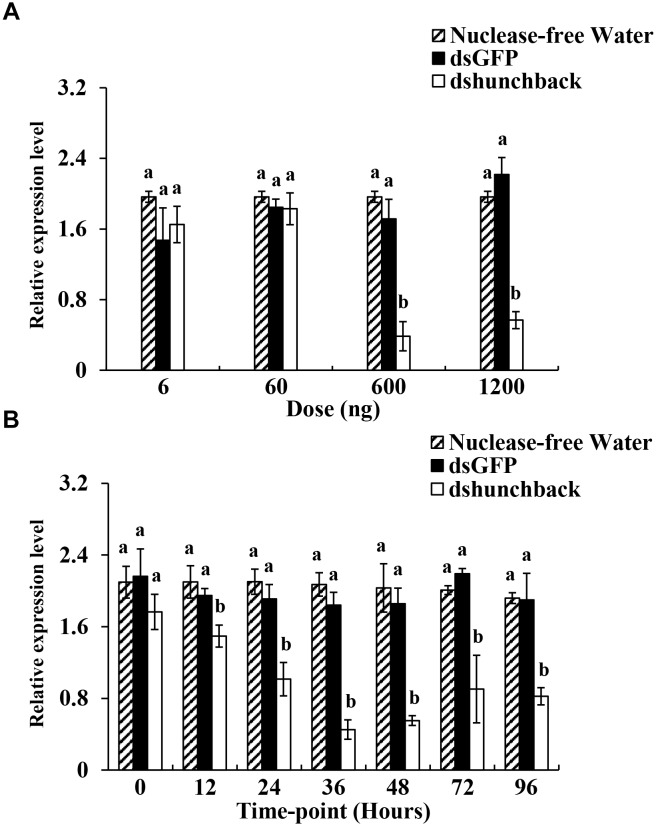
Relative expression of *hunchback* upon different ds*hunchback* doses and time-points after injection in *Acyrthosiphon pisum*. **(A)** Aphids were injected with 6, 60, 600, and 1200 ng, respectively. RNA was extracted after 36 h. **(B)** Silencing efficiency was detected at different sampling time-points (0, 12, 24, 48, 72, and 96 h) after the injection of 600 ng of dsRNA. Water and ds*GFP* was injected in the blank and negative control, respectively. RNA was extracted at each time-point. Each treatment contained four replicates analyzed by qPCR and four individuals were pooled in each replicate. Lower-case letters above each bar indicate significant differences among different treatments using analyses of one-way analysis of variance followed by Tukey’s test (mean ± SE; *P* < 0.05).

**FIGURE 2:**
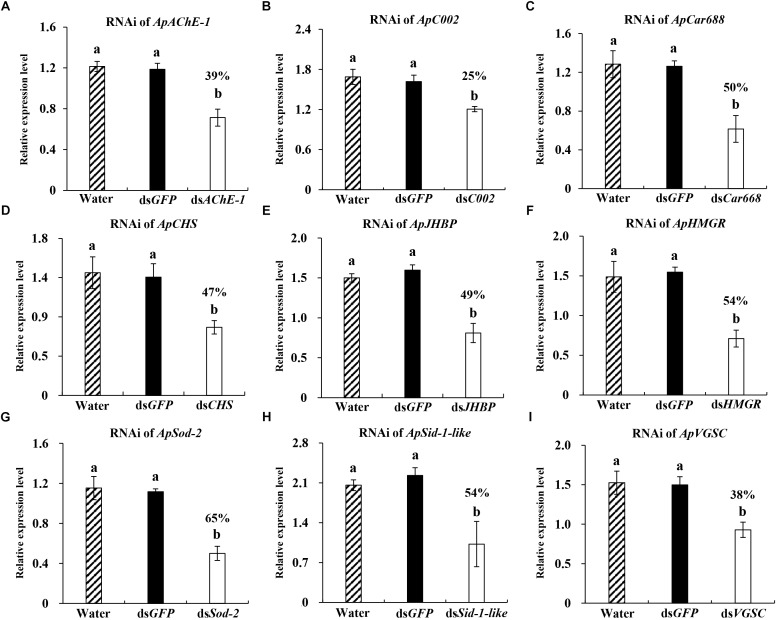
Relative expression of different target genes in *Acyrthosiphon pisum* at 36 h post injection of 600 ng dsRNA. **(A–I)** Water and ds*GFP* was injected in the blank and negative control. Each treatment contained four replicates analyzed by qPCR and four individuals were pooled in each replicate. Lower-case letters above each bar indicate significant differences among different treatments using analyses of one-way analysis of variance followed by Tukey’s test (mean ± SE; *P* < 0.05).

**FIGURE 3:**
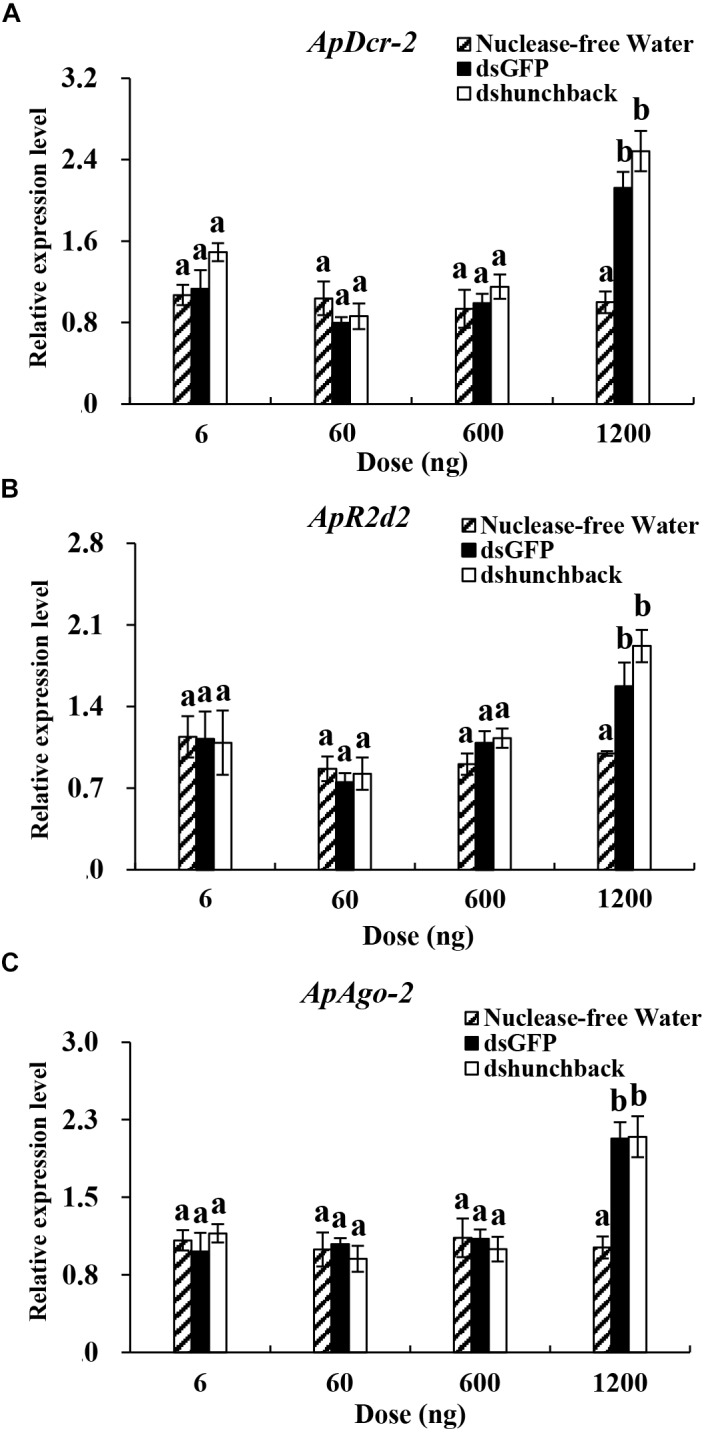
Relative expression of core siRNA pathway components (*ApDcr-2, ApR2d2*, and *ApAgo-2*) in *Acyrthosiphon pisum* at 36 h after the injection of different doses of dsRNA. **(A–C)** Aphids were injected with 6, 60, 600, and 1200 ng of dsRNA. Water was injected in the negative control. Each treatment contained four replicates analyzed by qPCR and four individuals were pooled in each replicate. RNA was extracted after 36 h. Lower-case letters above each bar indicate significant differences among different treatments using analyses of one-way analysis of variance followed by Tukey’s test (mean ± SE; *P* < 0.05).

**FIGURE 4:**
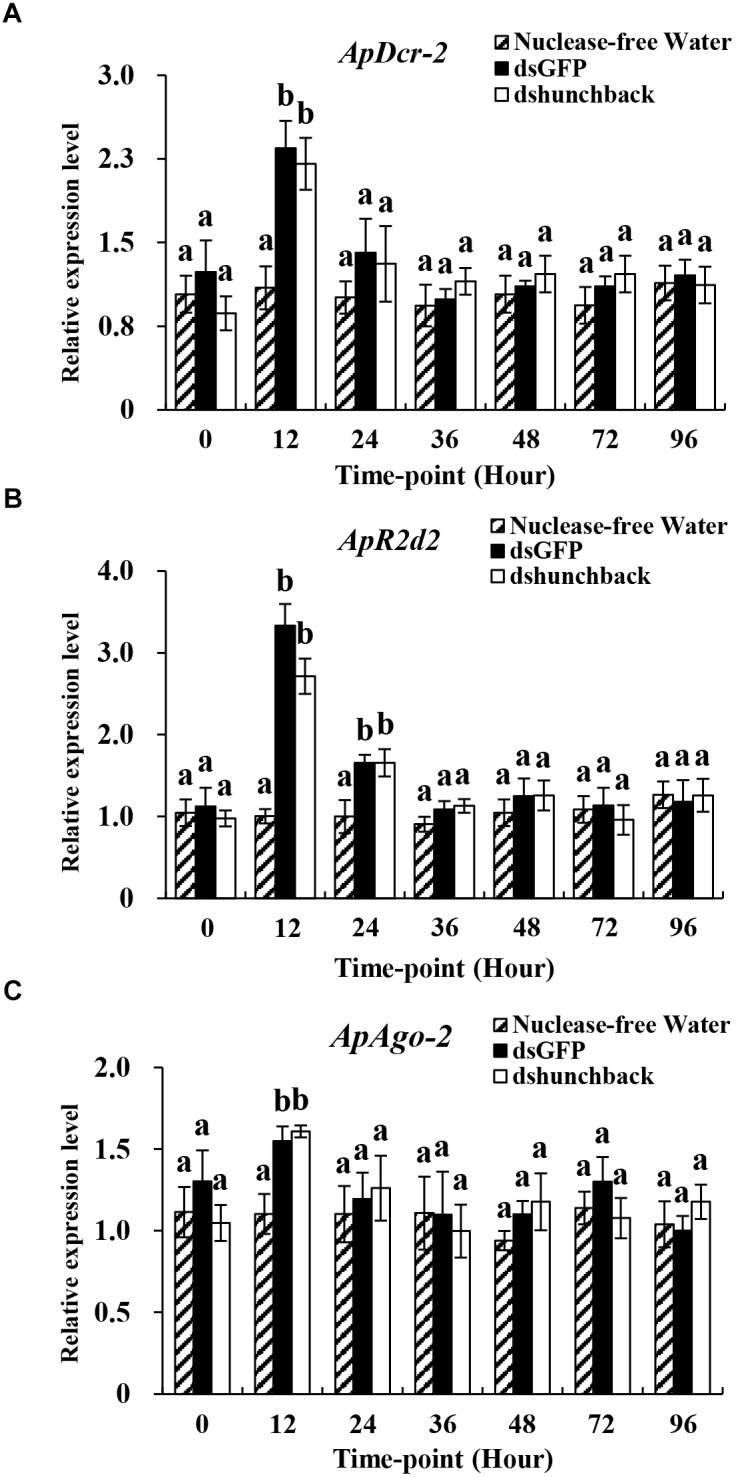
Relative expression of core siRNA pathway components (*ApDcr-2, ApR2d2*, and *ApAgo-2*) in *Acyrthosiphon pisum* at different time-points after the injection of 600 ng dsRNA. **(A–C)** Water was injected in the negative control. Each treatment contained four replicates analyzed by qPCR and four individuals were pooled in each replicate. Lower-case letters above each bar indicate significant differences among different treatments using analyses of one-way analysis of variance followed by Tukey’s test (mean ± SE; *P* < 0.05).

**FIGURE 5:**
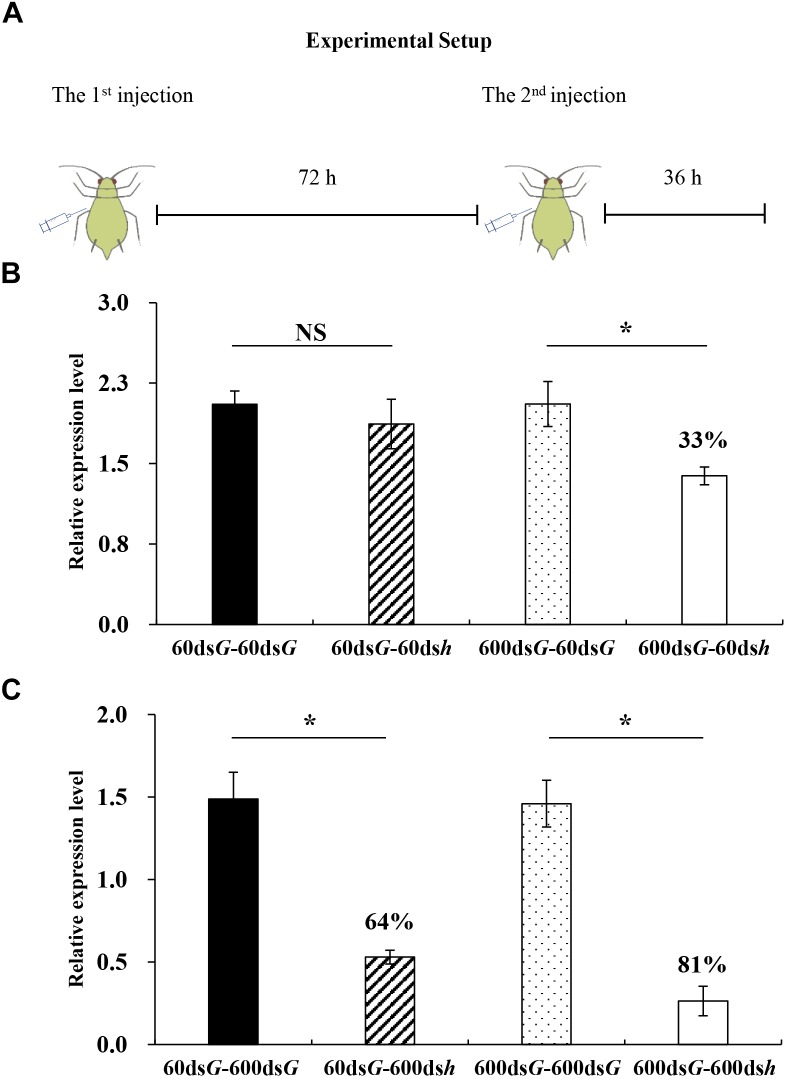
Silencing efficiency induced by a pre-injection of dsRNA. **(A)** Overview of the experimental setup. **(B)** Each bar represents a different treatment: 600ds*G*-60ds*G* = injection with 60 ng ds*GFP* after injection with 600 ng ds*GFP*; 600ds*G*-60ds*h* = injection with 60 ng ds*hunchback* after injection with 600 ng ds*GFP*; 60ds*G*-60ds*G* = injection with 60 ng ds*GFP* after injection with 60 ng ds*GFP*; 60ds*G*-60ds*h* = injection with 60 ng ds*hunchback* after injection with 60 ng ds*GFP*; **(C)** Each bar represents a different treatment: 60ds*G*-600ds*G* = injection with 60 ng ds*GFP* after injection with 600 ng ds*GFP*; 60ds*G*-600ds*h* = injection with 60 ng ds*GFP* after injection with 600 ng ds*hunchback*; 600ds*G*-600ds*G* = injection with 600 ng ds*GFP* after injection with 600 ng ds*GFP*; 600ds*G*-600ds*h* = injection with 600 ng ds*GFP* after injection with 600 ng ds*hunchback*. Each treatment contained four replicates and four individuals were pooled in each replicate. RNA was extracted after 108 h. After that, *hunchback* expression was analyzed by qPCR. ^∗^Indicate significant differences between control and treatment using a Student’s *t*-test (mean ± SE; *P* < 0.05).

The specificity of the qPCR primers was checked using a melting curve, and the PCR efficiency (Supplementary Table S2) was evaluated using a standard curve. For every experiment in this study, the qPCR analysis was based on four biological replicates (cDNA samples) for each treatment and each biological replicate was the result of pooling four individual aphids. For each biological replicate, two technical replicates were included on the qPCR plate. Furthermore, each experiment was also entirely repeated twice at a different time. qPCR was performed in a 96-well plate CFX Connect Real-Time System and analyzed by the Bio-Rad CFX manager software (Bio-Rad, Singapore). The qPCR was performed with a 10 μL reaction mixture containing 5.0 μL of NovoStart SYBR qPCR SuperMix (Novoprotein, Shanghai, China), 0.5 μL of cDNA template, 0.5 μL of each specific primer, and 3.5 μL nuclease-free water. PCR amplifications were performed with the following cycling conditions: 95°C for 2 min, followed by 39 cycles of 95°C for 15 s and 60°C for 30 s. At the end of each PCR run, a melting curve analysis was performed (from 60°C to 95°C in 0.5°C increments with a hold time of 5 s for each read). Two reference genes, *Rps20* and *EF1α*, were used to normalize the expression levels of targeted genes through qBase+ (Hellemans et al., 2007) based on the 2^-ΔΔCT^ method. *Rps20* and *EF1α* are stable reference genes in pea aphids that have been used in published papers (Chen et al., 2016; Chung et al., 2018). In addition, we examined the distribution of the Cq value in different target gene treatments (Supplementary Figure S3A) and determined the stability of reference genes by BestKeeper (Supplementary Figure S3B) (Pfaffl et al., 2004). We confirm that *Rps20* and *EF1α* were the relatively stable genes for various target genes. The reference genes in each plate were used to normalize the data and inter-run controls were added to allow plate comparison. Statistical differences were analyzed using one-way analysis of variance followed by Tukey’s test (Figures 1–4) and a Student’s *t*-test (Figure 5).

## Results

### Identification of an Optimal dsRNA Concentration and a Temporal Analysis of Gene Silencing Efficiency Targeting the *hunchback* Gene by Injection

To obtain an overview of the gene silencing efficiencies for dsRNA in pea aphids, four different doses of ds*hunchback* doses (6,60,600 and 1200 ng) were administered by injection to adult aphids. Two controls, ds*GFP* and RNA-free water, were used in the bioassay. As shown in Figure 1A, significant silencing of *Aphunchback* at 36 h post-injection (hpi) was achieved using 600 ng and 1200 ng of ds*hunchback*. Both amounts resulted in an ∼70% reduction in expression. To further evaluate the temporal evolution of the gene silencing efficiency in pea aphids over time, 600 ng of ds*hunchback* was injected and the gene expression was analyzed at seven different time points after injection (0, 12, 24, 36, 48, 72, and 96 h). As shown in Figure 1B, the significant silencing of *Aphunchback* could be detected as early as 12 hpi, and the most significant silencing efficiency (74%) was observed at 36 hpi. Moreover, gene silencing could be detected until the last analyzed time point, which was 96 hpi. In summary, based on our experimental conditions, optimal gene silencing in pea aphids for the *hunchback* gene was achieved by the injection of 600 ng dsRNA, and the greatest mRNA depletion rate was detected at 36 hpi.

### Evaluation of Pre-defined Gene Silencing Conditions Using Different Potential Target Genes Having Variable Functions

After ascertaining the optimal gene silencing conditions for the *hunchback* gene (600 ng dsRNA and 36 h after dsRNA injection), we aimed to determine whether the same gene silencing conditions could be used to obtain significant RNAi effects while targeting a wide range of other potential target genes having variable functions. For this, we selected *ApAChE-1, ApCar668, ApCHS, ApC002, ApHMGR, ApJHBP, ApSid-1-like, ApSod-2*, and *ApVGSC*. Among these genes, *ApAChE-1, ApCar668, ApCHS, ApHMGR, ApJHBP, ApSid-1-like, ApSod-2*, and *ApVGSC* were used as targets for RNAi in the pea aphid for the first time. A significant silencing level was achieved for all nine targeted genes, ranging from 25 to 65% transcript depletion (Figure 2). In detail, RNAi targeting of *ApAChE-1, ApCar668, ApCHS, ApC002, ApHMGR, ApJHBP, ApSid-1-like, ApSod-2*, and *ApVGSC*, resulted in 39, 50, 47, 25, 54, 49, 54, 65, and 38% downregulation, respectively. Thus, 600 ng dsRNA and 36 hpi are useful conditions for achieving the functional silencing of various genes in the pea aphid, even though the silencing efficiencies of the different genes varied.

### Evaluation of the Expression of Core RNAi Components in Pea Aphids Following the Administration of Different Amounts of dsRNA

The expression levels of three core RNAi components, *ApDcr2, ApR2d2*, and *ApAgo2*, were evaluated under two experimental conditions. In the first experiment, four different doses of ds*hunchback* treatments (6, 60, 600, and 1200 ng) were administered to adult pea aphids by injection and then the expression levels of the RNAi core genes were evaluated at 36 hpi (Figure 3). In the second experiment, the expression levels of the RNAi core genes were evaluated at seven different time points after injection (0, 12, 24, 36, 48, 72, and 96 h) with 600 ng of ds*hunchback* (Figure 4).

In the first experiment, the expression levels of *ApDcr2, ApR2d2*, and *ApAgo2* significantly increased after the injection of 1200 ng of ds*hunchback* or ds*GFP* compared with the injection of RNA-free water. For *ApDcr2*, 2.3-, and 2.5-fold increases in expression were observed in the ds*GFP* and ds*hunchback* treatments, respectively, (Figure 3A). Ds*GFP* and ds*hunchback* injection also led to the increased expression of *ApR2d2* (1.6- and 1.9-fold, respectively; Figure 3B) and *ApAgo2* (∼2.1-fold for both treatments; Figure 3C). In the second experiment, increased expression levels of *ApDcr2, ApR2d2*, and *ApAgo2* were detected as early as 12 hpi. At this time point, the expression levels of *ApDcr2* increased 2.4- and 2.2-fold following the injection of ds*GFP* and ds*hunchback*, respectively, (Figure 4A). For *ApR2d2*, we observed 3.3- and 2.7- fold increases for ds*GFP* and ds*hunchback*, respectively, (Figure 4B). In addition, the expression level of *ApR2d2* was significantly reduced at 24 hpi. For *ApAgo2*, the expression levels increased 1.5- and 1.6-fold at 12 hpi with ds*GFP* and ds*hunchback*, respectively, (Figure 4C).

### Gene Silencing Efficiency Can Be Improved by Priming the RNAi Machinery Through Pre-exposure to dsRNA

To explore whether an increase in the transcript levels of *ApDcr2, ApR2d2*, and *ApAgo2* could lead to an increase in the RNAi efficiency, we evaluated the effects of pre-injecting dsRNA into adult pea aphids then later verifying the gene silencing efficiency following a second injection. Either 60 ng or 600 ng of ds*GFP* was pre-injected into aphids, followed 72 h later by a second injection of 60 ng or 600 ng of ds*hunchback*, which acted as a reporter gene. The ds*GFP* was used for the second injection in the control (Figure 5A). The injection of 60 ng of ds*hunchback*, following the pre-injection of the 600 ng ds*GFP*, led to the significant silencing of the target gene by 33% at 36 h after the second injection (Figure 5B). In our earlier experiments, the injection of this dose did not successfully lead to gene silencing (Figure 1A). Similarly, after the pre-injection of 600 ng ds*GFP*, the injection of 600 ng of ds*hunchback* induced a significant silencing of the target gene by 81% at 36 h after the second injection (Figure 5C). The silencing was greater than that induced by 600 ng ds*hunchback* after pre-exposure to 60 ng of ds*GFP*. Moreover, the pre-injection of 60 ng of ds*GFP* may not result in a strong enough priming of the RNAi machinery (Figure 5B).

## Discussion

The functionality and efficacy of RNAi, as a functional genomics tool or a potential strategy for aphid pest control, are still unclear. While several successful RNAi experiments have been reported in this species (Mutti et al., 2006; Jaubert-Possamai et al., 2007), others have reported difficulties achieving significant gene silencing and the variability of RNAi efficiency (Christiaens and Smagghe, 2014; Yu et al., 2016). Furthermore, several studies have shown that efficient RNAi can be achieved in several other Hemiptera. For example: In second-instar *Euschistus heros*, 27.4 ng dsRNA injection reduced the *EhATPase A* and *Ehact-2* transcript levels by 90% and 59%, respectively, (Castellanos et al., 2018). In third-instar *Nilaparvata lugens* nymphs, the transcript levels of *NlCaM* and *NlCaM1* at 4 days after injection of 50 ng dsRNA were reduced by 67.9% and 62.8%, respectively, (Wang et al., 2018). Finally, in *Nephotettix cincticeps*, the expression levels of *NcSP75* had decreased to 0.2–0.5% of those of the control group at 2, 4, and 8 d after a 9 ng dsRNA-injection (Matsumoto and Hattori, 2018).

To gain a better insight into RNAi in aphids, we investigated the optimal injected dsRNA dose to achieve efficient RNAi in the model and pest pea aphid. Additionally, we investigated the temporal changes in the target gene transcript level after injection and the association between RNAi efficiency and the expression levels of core RNAi machinery components.

Information about dose- and time-dependent gene silencing efficiencies are crucial in determining a general image of RNAi sensitivity levels in targeted organisms. To obtain this information in the pea aphid, we first investigated the gene silencing efficiencies of four different dsRNA doses (6, 60, 600, and 1200 ng per insect) at 36 hpi. As a target gene, we initially chose the gap gene, *Aphunchback*, which is a key regulator in the anteroposterior patterning of insects and has been successfully silenced in two aphids species, *A. pisum* (Mao and Zeng, 2012) and *M. persicae* (Mao and Zeng, 2014) through plant-mediated RNAi. To further demonstrate the suitability of this target gene for these experiments, the expression of *Aphunchback* in the controls (injection with nuclease-free water) was found to be stable among all tested time points (Figure 1B) and did not lead to a strong lethality in the treated aphids (Supplementary Figure S2). Significant gene silencing could be achieved by injecting a dsRNA dose of 600 ng. The greater dose of 1200 ng dsRNA did not result in more efficient gene silencing.

The dsRNA dose used in previous *hunchback* silencing experiments in aphids cannot be compared with those used in this study, because the quantities of dsRNA actually ingested in the feeding experiments are unknown. However, the silencing of other genes by the injection of dsRNA in aphids was successful at the 10–460 ng dsRNA range. For pea aphids, the dsRNA dose delivered by injection in successful RNAi experiments ranged from 50 to 460 ng (Yu et al., 2016), suggesting that the necessary dose varied depending on the target gene. To determine whether the optimal dose found in this study could be used for a wide range of target genes, we tested nine other targets (*ApAChE-1, ApCar668, ApCHS, ApC002, ApHMGR, ApJHBP, ApSid-1-like, ApSod-2*, and *ApVGSC*) and successfully knockdown each gene. The silencing levels among these genes ranged from 25 to 65%, and we roughly divided these targets into two groups. The first, *ApCar668, ApCHS, ApHMGR, ApJHBP, ApSid-1-like*, and *ApSod-2* showed a silencing efficiency of 50–65%, while *ApAChE-1, ApVGSC*, and *ApC002*, showed silencing efficiencies of 25–39%. This variability could result from the latter three genes being highly tissue-specific. *ApAChE-1* and *ApVGSC* are mainly expressed in the central nervous system (Kawada et al., 2009; Mou et al., 2017), in which the neural lamellae and nerve sheaths of insects together constitute the blood–brain barrier to isolate the brain and hemolymph. *ApC002* is highly expressed in salivary glands and plays an important role during feeding in aphids (Mutti et al., 2006; Coleman et al., 2015). The successful silencing of this gene by feeding dsRNA was observed earlier in the pea aphid (Mutti et al., 2006), but later attempts to reproduce this result failed (Christiaens et al., 2014). This may have resulted from the involvement of strain-specific factors that could influence RNAi efficiency. In the current study, using a 600 ng-dsRNA dose, *ApC002* was successfully silenced but still showed the lowest silencing efficiency among the nine tested genes. In summary, while the 600-ng dsRNA dose is relatively high compared with the doses used in many earlier studies in the pea aphid, could be successfully used to knockdown a wide range of genes.

Another important issue regarding RNAi efficiency is the duration of the knockdown effect. In many insect species, gene expression can recover back to normal levels relatively quickly. For example: in *Cylas puncticollis*, the silencing of *Lac2* was observed to last for 10 d (Prentice et al., 2015). In third-instar nymphs of *A. pisum*, the silencing of *Cathepsin-L* after the injection of 322 ng dsRNA was found to last for 120 h (Sapountzis et al., 2014). Thus, looking at the temporal development of the silencing signal could deliver vital information for future experimental designs and, especially, the detection of gene knockdowns at the transcript level. Increased knowledge of the duration of gene silencing can also be applied by crop protectionists that are using RNAi as a pest management tool. In this study, gene silencing could be detected at the transcript level as early as 12 hpi with 600 ng of ds*hunchback*, indicating efficient gene silencing. The silencing of the targeted gene persisted until the last observed time point in this study (96 hpi).

In insects, the siRNA pathway evolved as an antiviral mechanism to combat viral infections (Gammon and Mello, 2015). Specifically, by sensing virus-related dsRNAs, including viral dsRNA replication intermediates, this antiviral mechanism is activated, and the dsRNA is processed by Dcr-2. With a viral infection, the RNAi machinery’s activity could be enhanced to combat the infection, for example, through the upregulation of the core components *Dcr-2, R2d2*, and *Ago-2* (Marques et al., 2013; Niu et al., 2016). In addition, non-specific dsRNA can also act as a trigger to induce the antiviral activity, as well as the RNAi activity, in certain insect species (Flenniken and Andino, 2013; Cappelle et al., 2016). In this study, we explored the possible association between RNAi efficiency and the expression levels of core RNAi machinery components in the pea aphid. Same as *Aphunchback*, the expression of *ApDcr-2, ApR2d2*, and *ApAgo-2* was stable among all tested time-points in non-dsRNA treatment. All three tested RNAi core machinery components, *ApDcr-2, ApR2d2*, and *ApAgo-*2, showed significant upregulation at 36 hpi with 1200 ng dsRNA. Intriguingly, the upregulation of these three core RNAi components could already be observed at 12 hpi with 600 ng dsRNA. This upregulation only lasted for a short period though, and was not observed at 24 hpi for *ApDcr2* and *ApAgo2* and at 36 hpi for *ApR2d2*. Furthermore, the upregulation that was observed here was much less pronounced than that reported for the tobacco hornworm *Manduca sexta*, in which, depending on the tissue, a 79- to even 395-fold upregulation of Dcr-2 was observed after the injection of 1 μg dsRNA (Garbutt and Reynolds, 2012). In the German cockroach *Blattella germanica*, which is sensitive to RNAi, a fivefold upregulation of Dcr-2 was reported after the injection of 400 ng dsRNA. Additionally, injecting 4000 ng did not cause a greater upregulation than 400 ng, and 40 ng dsRNA did not result in any upregulation of Dcr-2 (Lozano et al., 2012). Previously, in the pea aphid, Christiaens et al. (2014) could not detect any upregulation of these RNAi core genes. However, they only injected 50 ng dsRNA, which might not be enough to cause a significant upregulation. Furthermore, they only investigated the core genes’ expression levels at 6 and 24 h after injection. Based on the results in this research, the window of upregulation is relatively narrow, which means they could have missed any potential effects. Thus, the dsRNA delivered to the aphids could significantly induce the expression of core RNA machinery genes as early as 12 h after dsRNA injection, and a greater dsRNA dose could prolong this upregulated gene expression. Interestingly, the injection of dsRNA could prime the RNAi machinery and increase the RNAi efficiency upon a second dsRNA administration. Injecting 60 ng of ds*hunchback* led to 33% gene silencing after a pre-injection of 600 ng of non-specific dsRNA, while injecting 60 ng of ds*hunchback* without prior priming of the RNAi machinery was not enough to elicit gene silencing. While this could be directly linked to the upregulation of the RNAi core genes, it could also relate to changes in the gene expression levels of other RNAi-related genes. However, no details are presently known and further research is required. In this experiment, we decided to perform the follow-up dsRNA injection at 72 h after the first injection, even though the core genes were found to be upregulated at 12 h after injection. We considered that a longer time frame was necessary between both injections, to allow for protein synthesis and to make sure the upregulation was represented at the protein level as well. In addition to investigating the mechanisms, our observations generated an intriguing question based on many plant viruses having dsRNA structures and aphids being well-known vectors of many of these viruses: what is the impact of this viral load and (pre-) exposure to these dsRNA structures on aphid immunity and RNAi efficacy when using RNAi-based insecticides?

In this study, we focused on the induced effects caused by injection of exogenous dsRNA. In the context of potential RNAi-based pest management of aphids, other delivery methods will have to be considered and further investigated. Indeed, there are multiple methods for dsRNA delivery, including injection, feeding, soaking (Yu et al., 2013), and topical application (Pridgeon et al., 2008; Killiny et al., 2014). Among them, feeding and injection are the most common methods. Injection is considered to be the most effective and direct way for screening potential target genes. However, it is only limited to be used in a laboratory setting and cannot be applied as a strategy in pest control. Compared with injection, plant-mediated (feeding) RNAi is a more practical approach to deliver dsRNA. Nevertheless, studies have shown that degradation by non-specific dsRNases in the saliva or midgut of insects could be an obstacle in achieving efficient RNAi-silencing by oral ingestion of dsRNA (Christiaens et al., 2014; Prentice et al., 2017; Castellanos et al., 2018), resulting in ineffective gene silencing. Of course, a continuous high supply of dsRNA, for example through transgenic plants, could help to avoid such issues. In addition, utilization of the synergy of RNAi with plants and microbes presents a potentially effective strategy in enhancing dsRNA delivery, efficiency, specificity, as well as reducing the risk in developing resistance to RNAi (Niu et al., 2018).

## Conclusion

In conclusion, we provided a general image of dose- and time-dependent gene silencing efficiencies in pea aphids. The RNAi pathway could be primed by non-specific exogenous dsRNA, leading to an enhanced gene silencing efficiency in pea aphids. The results presented in this study can be exploited in the development of more efficient RNAi bioassays for pea aphids. And while this study focuses on delivery by injection, it contributes important information on the RNAi mechanism and efficiency in aphids which can be valuable for further studies and optimization of RNAi through oral delivery in aphids.

## Ethics Statement

The research project was conducted on invertebrate species that are not subjected to any specific ethical issue and legislation.

## Author Contributions

CY, GS, JN, and J-JW designed the research. CY performed all of the experiments with the help of XA, Y-DJ, B-YD, and FS. J-JW provided the materials. CY, XA, Y-DJ analyzed the data. CY, OC, CT, GS, and J-JW wrote the paper.

## Conflict of Interest Statement

The authors declare that the research was conducted in the absence of any commercial or financial relationships that could be construed as a potential conflict of interest.

## Footnotes

1 https://www.ncbi.nlm.nih.gov/tools/primer-blast/index.cgi?LINK_LOC=BlastHome

## Supplementary Material

FIGURE S1 Size of experimental aphids. Size of adult aphids (*n* = 50) in this study were measured under a Leica M205 C microscope (Leica Microsystems, Buffalo Grove, IL, United States) using the software Leica Application Suite (LAS) (mean ± SE).

FIGURE S2 Cumulative mortality after twice injection. Number of deaths (*n* = 240) was recorded at each 12 h after injection until 108 h. The data was analyzed using Kaplan–Meier (Estimated value ± SE) to determine the mortality of aphids after injection.

FIGURE S3 Detection of the stability of the reference gene in different dsRNA treatments. **(A)** The distribution of Cq values of *Rps20* and *EF1α* was analyzed using Origin 9.0 (OriginLab, Northampton, Massachusetts, United States). **(B)** The stability of reference genes was determined by BestKeeper (Pfaffl et al., 2004).

TABLE S1 Accession numbers of RNAi targeted genes and RNAi core genes.

TABLE S2 Primers used for dsRNA synthesis and qPCR.
