# Supplementary material for: Induction of RNAi Core Machinery’s Gene Expression by Exogenous dsRNA and the Effects of Pre-exposure to dsRNA on the Gene Silencing Efficiency in the Pea Aphid (Acyrthosiphon pisum)
Source: Front Physiol. 2019 Jan 9;9:1906. doi: 10.3389/fphys.2018.01906 (PMC6333656; doi:10.3389/fphys.2018.01906)
Supplement: FIGURE S1 — Size of experimental aphids. Size of adult aphids (n = 50) in this study were measured under a Leica M205 C microscope (Leica Microsystems, Buffalo Grove, IL, United States) using the software Leica Application Suite (LAS) (mean ± SE). [file Data_Sheet_1.PDF]

Table S1. Accession numbers of *A. pisum* genes and RNAi core genes used in this study

Genes	GenBank No. (NCBI)
ApAChE-1	XM_008187580.2
ApCar668	XM_003241620.3
ApCHS	XM_016807983.1
ApC002	XM_001948323.3
ApHMGR	XM_016806644.1
Aphunchback	NM_001162510.1
ApJHBP	XM_016801586.1
ApSid-1-like	XM_008188194.2
ApSod-2	NM_001162681.2
ApVGSC	XM_008185139.2
ApEF1α	XM_001948705.4
ApRps20	NM_001162819.2
ApAgo-2	XM_001944817.3
ApDcr-2	XM_003240062.1
ApR2d2	NM_001162172.2

Table S2. Primers used for dsRNA synthesis and RT-qPCR

Fragment	Forward (5' to 3')	Reversed (5' to 3')	PCR Type and efficiency	Length (bp) of dsRNA
ds*AChE-1*	[a] GCCAGAATACACGGAGAAGC	[a] CTGCAGCGTTTTGTGGTCTA	[b] RT-PCR	455
ds*Car668*	[a] ATGTCATGTTTCCGGGCAT	[a] ACCTGATAGCATCCAAAGA	[b] RT-PCR	487
ds*CHS*	[a] CATTGTCACGTGGGGTACGA	[a] CTGATGTGGGACGCTACTGG	[b] RT-PCR	364
ds*C002*	[a] TGCAGCTGATGGAACTAACG	[a] CTCGTTTTCGGACGGTTTTA	[b] RT-PCR	365
ds*HMGR*	[a] TTAATGTTGGTGAAATCGAACCAT	[a] ACATTTTTGCTTCGCTAGCAGT	[b] RT-PCR	421
ds*hunchback*	[a] CATCACCGTACTCGATGCAA	[a] GGAGCCACTGAGTCTCTGCT	[b] RT-PCR	448
ds*JHBP*	[a] TAAATCGCTCGGTTGGCCT	[a] CCTGGTGTGTCTAAGGTTACG	[b] RT-PCR	402
ds*Sid-1-like*	[a] TTTGGTGTTTTGGCATTTGT	[a] GGCAGACCAAAATATCATAGCC	[b] RT-PCR	444
ds*Sod-2*	[a] ACGCAAAGCCATAGTCGTTC	[a] GGATCAAGGATTCCGATGAC	[b] RT-PCR	459
ds*VGSC*	[a] TGTGTCACCAACGCTTCTTC	[a] CACCAGGTACGACAGCAAAA	[b] RT-PCR	415
ds*GFP*	[a] TGAGCAAGGGCGAGGAGCTG	[a] TCGATGCGGTTCACCAG	[b] RT-PCR	370
q*AChE-1*	ACCAAACACATGCGTTCAAA	CAGACCCGGAGTAAAATCCA	RT-qPCR [c] *E*=108.6	
q*Car668*	CTTGTGAACATTGCCCGTGA	GAACCGAACCTCTTGTGACG	RT-qPCR [c] *E*=103.3	
q*CHS*	ACTGGGCGAGGACGGTATC	ACTCTTCGGCGGCTTTCTT	RT-qPCR [c] *E*=104.0	
q*C002*	AGGAAGAAGCGTCTGTCGAA	AGTAATGGGCGTTCTGGTTG	RT-qPCR [c] *E*=107.5	
q*HMGR*	GTGGAAATCAGGCCTTTTGA	CAACACCCCTCTCTGGGTTA	RT-qPCR [c] *E*=109.4	
q*huncback*	AAAACCGGCACAACCCGTAT	GGAAGCAGGCATCTGCAACA	RT-qPCR [c] *E*=96.1	
q*JHBP*	ACCATGCCACAGGATATGGAA	TCATGACCATCAGGATCAGCAA	RT-qPCR [c] *E*=106.5	
q*Sid-1-like*	TGGGATTGGCCATAAGTCAT	CGTTCTTGATTGAGCTGCAC	RT-qPCR [c] *E*=100.9	
q*Sod-2*	ACAATGGCACCCAAAATCAT	TTGGTCACGTCTCCCTTCTC	RT-qPCR [c] *E*=102.9	
q*VGSC*	TATCCTTAGCGGGTTTGGTG	CGCTGGAAGTGACATTGCTA	RT-qPCR [c] *E*=90.8	
q*EF1α*	CTGTGCTTATTGTCGCTGCT	TCGCTGTATGGTGGTTCAGT	RT-qPCR [c] *E*=99.7	
q*Rps20*	AAGTGTGTGCTCCGAGATGA	CAGCAATGACACCGGGTTC	RT-qPCR [c] *E*=98.6	
q*Ago-2*	CAGGCACCACAGAAACAAAA	AATTTGTTGACCGCCTTGAG	RT-qPCR [c] *E*=94.3	
q*Dcr-2*	TGGTCTCAACAGCAATGGAA	AATACGGGGACGTTCATCAG	RT-qPCR [c] *E*=96.9	
q*R2d2*	AGAAGGACAAGCCCATGCTA	TGGATTCCCTTCCATTACCA	RT-qPCR [c] *E*=104.4	

[a] The gene-specific parts of the primer are listed. These are preceded by the T7 adaptor TAATACGACTCACTATAGGG for dsRNA synthesis.

[b] These primers were used in RT-PCR for dsRNA synthesis.

[c] These primers were used in RT-qPCR for mRNA level detection. *E*: primers' amplification efficiency (%).

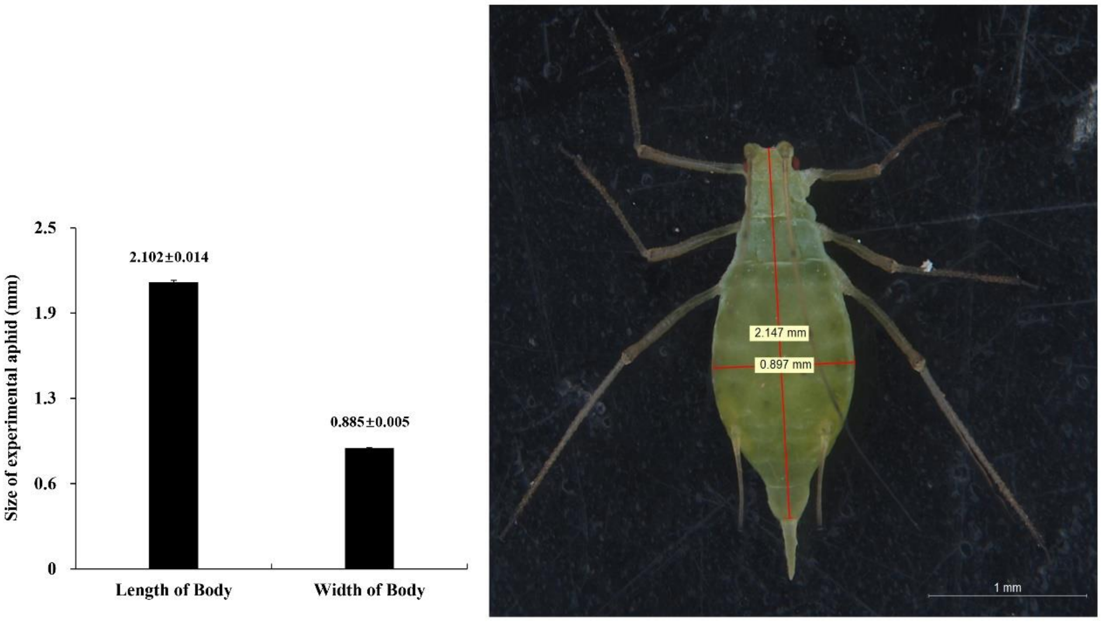

Figure S1. Body size of aphids used in this study

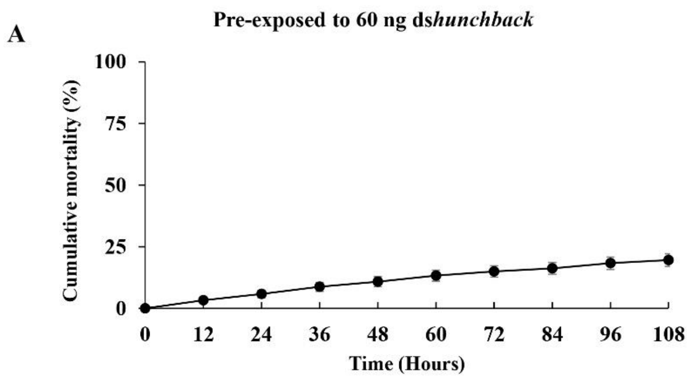

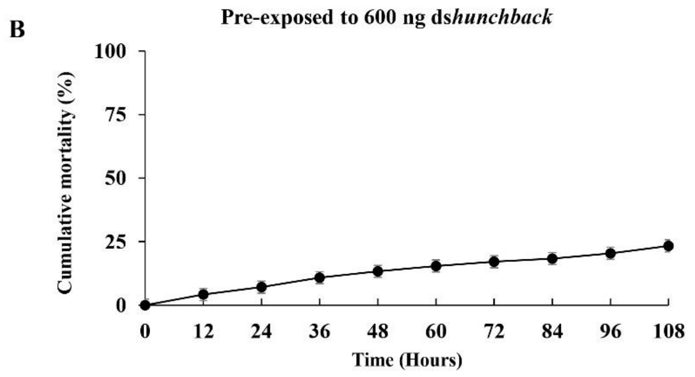

Figure S2. Cumulative mortality after the second injection

A

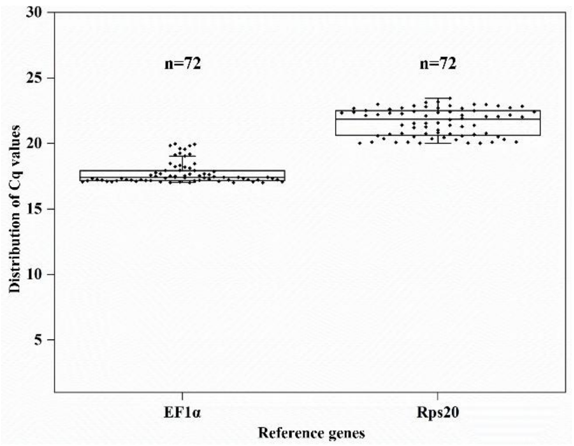

B

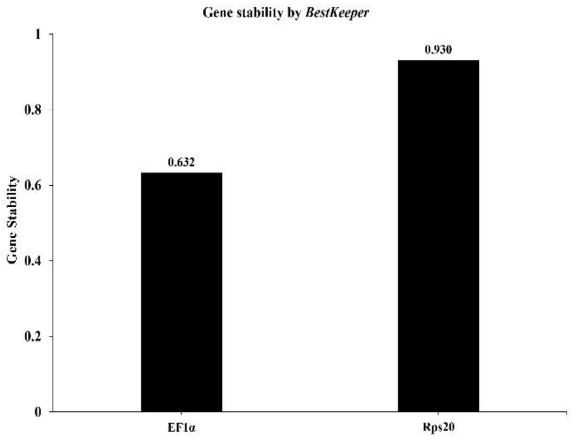

Figure S3. Detection of the stability of the reference gene in different dsRNA treatments. (A) The distribution of Cq values of *Rps20* and *EF1α* was analyzed using Origin 9.0 (OriginLab, USA). (B) The stability of reference genes was determined by BestKeeper (Pfaffl *et al*., 2004).
